# 

**Published:** 1891-01

**Authors:** 


### TABLE OF CONTENTS.

### 1891.

PAGE

Against Alcohol....................230
Aluminum ..........................211
Ammonia as a Power.................232
A Boy Mesmerist....................179
Ancient Breathing Exercises........ 66
A Chinese Custom...................132
A Case of Trance...................206
A "Cold in the Head"............... 56
A Dying Wife's Dream...............235
A Fact Stranger than Fiction....... 74
All Forms of Life Cellular.........208
A Finnish Bath..................... 62
Alcohol in Patent Medicines........234
A Laugh Producing Plant............259
A Maxim............................ 48
A Monster to be avoided............197
A New System of Cure............... 37
A Narrow Chance....................203
Alcoholic Prescriptions ........... 68
African Pygmies....................174
A Hint on Economy .................275
An Insect Worth Millions ..........273
A Remarkable Case.................. 31
Almost Time........................144
A Truth Worth Considering..........166
A Veteran's Manner of Life......... 88
A Word with our Readers ...........193
Austrian Women.....................227
Ammonia in the Household...........260

Baldness........................... 40
Bear Skin .........................276
Bronchitis.........................108
Bugs and Hydrophobia...............251
Bathing a Sanitary Need............101
Bright's Disease...................112
Blindness in New-Born Infants......138
Bacteria in Milk...................250
Bow-Legs...........................162

Care of Clothing ..................278
Children........................... 58
Cancer.............................200
Celery.............................212
Candies and Sweets................. 80
Celery ............................271
Cheese as an Article of Diet.......218
Cleaning Windows and Paint.........277
Cleanliness and Health.............171
Concerning Boils................... 36
Consumption Cured by Subcutaneous Injections............................ 1
Coffee Drinking.................... 8
Clairvoyance Extraordinary......... 42
Cold Food..........................253
Capacity for Food of Infants.......106
Cold Food in Winter................ 82
Cure for Round Shoulders...........232
Cure for Burns.....................139
Coffee; its Use and Abuse..........147
Convulsions ....................... 34
Convulsions in Children ...........157
Curiosities of the Vegetable Kingdom........194
Children's Teeth...................244
Children's Teeth...................184

Dietary............................ 17
Driver Ants........................188
Disinfection by Sulphur............ 13
Dressing for the Open Air..........108
Discovery in the Necropolis of Thebes.......258
Dining in Lapland.................. 21
Diseases of the Rich...............242

PAGE

Deadly Poisoned Arrows............. 43
Don't Shut up the Windows..........161

Executions by Electricity..........217
Electric Belts and Pads............233
Extraordinary Case of Somnambulism.178
Eucalyptus Disinfectant............228
Exercise for Girls ................220
Elephantine Intelligence...........165
Epidemic Influenza.................159
Every man his own Tailor...........115
Effect of Climatic Changes......... 60
Edith Thomas's Blindness........... 57
Eight Weeks Without Food...........185

Fainting...........................156
Faith Cure and Cancer..............139
Food Exhibition....................239
Food for Dyspeptics ...............270
Formation of Chalk.................189
Fidgety Parents....................156
From the Dark Ages.................256
Five Ways to Cure a Cold........... 36
For a Burn.........................248

Giants ............................154
Giving Animal Food to Infants......107
Good Advice to Girls...............243
Good Health........................145
Gas Poisoning Hints................ 87
Good Tea...........................253
Good to Remember...................279

Habits of Eating...................266
Headache and the Eyes.............. 34
Hay Fever..........................222
Hot Milk a Stimulant...............208
Hygiene of the Eye................. 63
How to Avoid Choking...............210
How to Treat the Ear .............. 9
How Tobacco Smoking injures Yale Students.171
How to Make a Poultice.............253
How to Speak.......................225
How to Grow Plump..................224
How to Cook a Husband .............238
How to be Beautiful ...............140
How to Look Well .................. 30
How to Treat One in a Faint ....... 88
How to Keep the Baby Well .........182
Harvey's Wonderful Power .......... 20
Hot Water Cures ...................247

If We Could Know...................192
Impure Air ........................ 56
In Contagious Diseases.............277
Infant Diet .......................280
Inflamed Eyelids...................109
Inflamed Eyelids...................221

Literary .22-46-71-94-118-142-167-191-216-240-283
La Grippe.......................... 97
Laughter...........................103
Look for the "Russian Itch"........166
Liver Spots........................ 81
Light Weight Bedding .............. 66
Life...............................261

Mosquitoes.........................139
Miscellaneous......19-41-67-91-116-140-164-188-212-236-261-281
Monotony in Farm Life..............114
Mental and Physical Work...........158
Marriage among New York Plutocrats.229

PAGE
Milk Impurities..........................248
Mole on Face..............................127
Medicinal Properties of Food................134
Mind Reading.............................. 25
Managing the Little Ones................... 86
Marriage with Drunkards.................... 82

Now and Then I Question Me.................. 72
Neatness in Dress at Home...................210
Necessity of Natural Sleep..................105
Nutritive Value of Foods.................... 60

Oatmeal as a Food..........................269
Overstrung Nerves..........................270
Oysters as Food............................ 85
Our Birth.................................. 24
Open Competitive Examination of M. D's...144
Over Eating................................106
Our Prison Systems......................... 49

Petrified in a Pit..........................183
Poisoned Air...............................126
Putting Away Winter Clothing...............125
Perspiring Feet............................122
Progress on the Congo......................254
Primitive Practitioners....................249
Pneumonia.................................. 64
Precious Stones of the Month...............131
Precious Stones; Early Notions regarding them....163
Professor Swing on Immortality............. 32

Ringworms and Eczema.......................135
Rules for Dyspetics........................246
Rules for Good Health...................... 79
Remarkable Psychic Transformation of Mary Vennum.................................... 61

Sunlight and Health........................161
Sleep and Health...........................169
Short and Tall Men.........................224
Stuttering Cured...........................140
Simple Diet the Safest.....................155
Spiritual Evidence.........................181
Sunday for the People......................247
Short Lived Beauty......................... 77
Sale of Tea in Russia......................162
Speed of the Electric Current..............213
Standards of Beauty........................180
Sponging out a Headache....................202
Statistics of Interest...................... 9
Sunny Rooms for Health.....................108
Sunlight...................................180
Stammering.................................245
Second Sight............................... 58
Small Things of Great Account.............. 93
Spring Tonics..............................160

The Age of Decay...........................198
The Ascending Spirit.......................137
The Bath................................... 81
The Baby...................................173
The Best Bread.............................251
The Brain..................................132
The Corset................................. 8

PAGE
The Corset Once More.......................127
The Costliest Gift......................... 96
The Dandelion..............................163
The Earth's Heat...........................274
The First Meerschaum Pipe..................213
The Fly a Carrier of Micro-organisms....... 10
The Finger Nails........................... 79
The Hygiene of Motherhood.................. 4
The Hope of Immortality.................... 14
The Hygiene of Motherhood.................. 26
The Hair...................................223
The Hygiene of Motherhood.................. 51
The Human Figure...........................110
The Hygiene of the Home.................... 73
The Infant Brain...........................133
The Intense Brilliancy of Lightning........276
The Land of Pluck..........................172
Telling the Truth for Fear of the "Cop"...150
The Mystery of a Lost Limb.................151
Too Many Meals............................. 35
The Medicinal Value of Onions..............272
Table Manners..............................226
The New Year............................... 1
The Next Volume............................265
Treatment of Fever......................... 18
The Poet...................................120
The Process of Digestion................... 98
The Retort.................................216
Tan Spots..................................176
The Sewage Farms of Berlin................. 15
To Take Out Paint..........................215
The Term Microbe........................... 38
Tendency to Physical Degeneration..........160
The Medicinal value of the Yellow Marigold 178
The Waste of the Household.................268
Talleyrand................................. 67
Thwarting the Divine Will..................181
Time.......................................168
The Use of Drugs........................... 40
The Value of Sleep for Women............... 83
Two Remarkable Spiders.....................111

Under the Earth............................199

Variety in Food............................123
Victor Hugo's Presage of Immortality.......209

Warts......................................177
Water as a Medicine........................205
Water and Health...........................243
Water at Meals............................. 33
What is a Cell?............................201
Weak Knees.................................124
Wedding Rings..............................273
Who Pulled the Bell-rope?.................. 12
What Rang the Telephone Bells?............. 39
Worth Remembering..........................204
Whitening the Hands and Face...............175
Weary......................................240
Worms......................................207
What the Danish Women Learn................129

Year-Old Babies............................ 84
Youth and Old Age..........................216

### LITERARY.

### SPECIAL.

We have instructed our bookkeeper to carefully revise our subscription list, and strike from the roll the names of all *delinquents*, as well as all subscribers whose paid up terms expired with the December issue. It is a little unpleasant to have unpaid bills, which have been running from two to four years, returned to us with the statement that the delinquent subscribed for only *one* year, and does not propose to *pay* for the years he has received the Journal *unordered.* This is a small matter in any individual case, but where it includes hundreds it amounts to a very considerable sum to be put down to profit and loss.

If any of our readers feel disappointed at not receiving the January number, they have only to renew their subscriptions from that date and receive it.

We have adopted the rule of terminating all subscriptions at the close of the pre-paid term, as the one most satisfactory to all parties.

Those who have heretofore continued to receive the Journal for the matter of two or three years without paying for it, will accept our humble apology for having occasioned them so much annoyance, with the compliments of the season, and a promise of speedy reform.

---

THE MONIST, a philosophical quarterly, issued by the Open Court Publishing Company, Chicago, bids fair to take a high place among our serial publications of the better sort. The January number is brilliant in instructive essays.

GOOD HOUSEKEEPING has become a monthly. It makes its first appearance in its new and attractive form as a New Year's offering, and a very acceptable one it is, too. There is none better of its kind extant, and if it meets with the favor it deserves, its financial career is bound to be one of entire success.

THE NEW METHOD IN CERTAIN CHRONIC DISEASES.—By W. E. Forest, B. S., M. D., M. L. Holbrook & Co., Publishers, 710 Broadway, New York.

"The older the physician grows, and the wider his experience, the more futile does he find mere drugs, and drugs alone, in the cure of chronic and sub-acute diseases." Such are the opening words of this admirable little treatise of 123 pp. Among the subjects treated of are Exercise, Diet, Dyspepsia, Biliousness, Kidney Diseases, Nervous Exhaustion, Catarrh, Hemorrhoids, Rheumatism and Corpulency. The methods are simple and doubtless effective, the more so as the poisonous effects of the old style drugging are supervened by a more rational system. The book will be found valuable, not only to physicians, but to those who have suffered long and patiently under their hands.

MEDICAL BULLETIN VISITING LIST, NO. 1.—This is a pocket memorandum book intended specially for physicians in active practice, with printed formulæ, guides, tables and prescriptions adapted to various diseases, ages and shades of disorders common to the ordinary run of patients. It will be found indispensable as a ready reference and visiting note book. Published and sold by F. A. Davis, 1231 Filbert street, Philadelphia.

We have received a treatise, or something of the sort, entitled

SILVER IN THE FIFTY-FIRST CONGRESS, with a SUMMARY OF THE U. S. COINAGE LAWS.

We shall make no attempt to review this monetary exposition. The truth is we have so little to do with money in any shape, that our opinion would be utterly valueless, yet we can always remember, within a million or two, the amount of our drawn and honored checks and this was more than one of our leading financiers could do a few days after the transaction. *His* opinion would be worth something.

Our thanks are extended to the authors who have favored us with the following publications:

IMPERFORATE AUDITORY CANALS.—By Seth S. Bishop, M. D., Chicago, Ill.

THE BACTERIOLOGICAL WORLD.—A monthly illustrated magazine for the study of micro-organisms and diseases of bacterial and parasitic origin. Paul Poquin, M D., V. M., &c., Editor, State University, Columbia, Mo., will be commenced the 1st. of January and continued in monthly issues thereafter. The subject is one of absorbing interest at this time. The frontispiece will consist of engraved likenesses of the two eminent discoverers in medicine, Pasteur and Koch.

THE TREATMENT OF THE MORPHINE DISEASE.—By J. B. Mattison, M. D., of the Home for Habitués, Brooklyn, N. Y. Reprint from the Therapeutic Gazette, of Sept. 1, 1890, 24 pp.

IN GORGEOUS ARRAY.—A number of our exchanges come to us in holiday dress, in honor of Christmas and the New Year. Among them we would mention the Churchman, Youth's Companion, Cottage Hearth, Baby Land and Our Little Men and Women.

We have also received a number of Illuminated Calendars for 1891, among them one very handsome in design and execution from that excellent serial, "The Canadian Queen," of Toronto, Canada, which we commend to our lady readers. One thing, however, troubles us. The calendar was labeled in staring capitals, "FOR THE EDITOR'S WIFE." Now, supposing the editor has no wife, would it be larceny for him to appropriate the gift ; or supposing there are two or three editors with wives, how would it be possible to make peace between them ? We cannot sub-divide the calendar any more satisfactorily than could the wise old patriarch the living baby of two claimants, and even if we should, one or the other would want all the springtime, or the summer, or the harvest season. On reflection, we conclude that the luckey *he* member into whose hands it fell, for the sake of peace, had best keep it.

THE CENTURY MAGAZINE.—Always so good there is no room for improvement.

THE HOME MAKER.—Rightly named.

LEND A HAND.—Excellent in its way and its way of the *best*.

TABLE TALK.—Full of good *things*.

GOOD HOUSEKEEPING.—No better.

Indeed, all our exchanges deserve particular mention, but we forbear for want of space. Good luck and a Happy New Year to every one of them.

ON THE MEDICATED AIR TREATMENT OF CONSUMPTION, ETC.—By Robert Hunter, M. D., New York City, the founder of the treatment, to the exposition of which this pamphlet of 32 pp. is devoted.

The author says in his preface that the aim of his treatise is to make plain three important facts. "*First*, the perfect curability of lung diseases ; *secondly*, the true cause of their fatality under ordinary medication, and *lastly*, the success which attends their proper treatment by medicated air and vapor.

PYOKTANIN IN DISEASES OF THE EYE, EAR AND THROAT.—By W. Cheatham, M. D., Specialist, etc., University of Louisville. A brief but comprehensive treatise, in which it is explained that Pyoktanin is a combination of coal tar extracts and signifies "pus-killer."

